# Effects of 8-hydroxyquinoline-coated graphene oxide on cell death and apoptosis in MCF-7 and MCF-10 breast cell lines

**DOI:** 10.22038/ijbms.2020.41277.9751

**Published:** 2020-07

**Authors:** Firoozeh Kheiltash, Kazem Parivar, Nasim Hayati Roodbari, Behnam Sadeghi, Alireza Badiei

**Affiliations:** 1 Department of Biology, Science and Research Branch, Islamic Azad University, Tehran, Iran; 2 Cancer Immunotherapy and Regenerative Medicine, Breast Cancer Research Center, Motamed Cancer Institute, ACECR, Tehran, Iran; 3 School of Chemistry, College of Science, University of Tehran, Tehran, Iran

**Keywords:** 8-Hydroxyquinoline, Apoptosis, Breast cancer, Cytotoxicity, Graphene oxide

## Abstract

**Objective(s):**

Breast cancer is a devastating disease related to women. The anticancer properties of 8-hydroxyquinoline (8HQ) and the increasing use of graphene oxide (GO), as a drug delivery system with anti-cancerous properties, led us to investigate the toxicity and apoptosis-induction capability of 8HQ-coated GO on breast cancer cells compared with normal breast cells.

**Materials and Methods:**

Breast cancer (MCF-7) and normal breast (MCF-10) cell lines were treated with several doses of 8-HQ-coated GO for 12, 24, and 48 hr. The toxicity of the nanocomposite was measured using MTT assay and the effect of the nanocomposite on cell apoptosis was determined by examining the expression of *P53, P21, Bax*, and *BCL2* genes, as well as Annexin-V /PI apoptosis assay.

**Results:**

There were significantly increased cell deaths in nanocomposite-treated MCF-7 breast cancer cells, compared with treated normal breast cells. Significantly increased expression of *P53, P21, *and *Bax* genes and reduced expression of *BCL2* gene were found in the treated breast cancer cell line compared with the normal cell line. Annexin-V/PI assay also illustrated significant induction of apoptosis in MCF-7 following nanocomposite treatment.

**Conclusion:**

Overall, 8HQ-coated GO has toxicity for breast cancer cell lines and one of the mechanisms through which this nanocomposite can induce cell death is the induction of apoptosis. With complementary *in vitro* and* in vivo* studies, this nanocomposite can be suggested as a nano-drug with anti-cancer properties.

## Introduction

Breast cancers in women are some of the major causes of death worldwide. Global cancer incidence is estimated at 18.1 million and about 9.6 million deaths in 2018 (1). Different therapies exist based on the type of cancer and its progression, including surgery, chemotherapy, radiotherapy, hormone therapy, and immunotherapy (2). Apoptosis is one of the main mechanisms to eradicate cancerous cells and inhibit this process by increasing anti-apoptotic proteins and decreasing pro-apoptotic proteins, resulting in the survival of cancer cells (3). That is why apoptosis has attracted a lot of attention in anti-cancer research. The *BCL2* protein family plays an important role in mitochondrial-dependent apoptosis. The members of this family are divided into two main groups of pro-apoptotic and anti-apoptotic proteins. The members of pro-apoptotic, especially Bax, play an important role in the onset of apoptosis (4). On the other hand, P53 protein is an important protein in inducing apoptosis in damaged or cancerous cells. The activated P53 binds to DNA and induces the expression of some genes, including P21 (5). The P21 protein by binding to the cell cycle complexes inhibits cell cycle and cell proliferation and acts as a stop signal in cell division. In contrast, anti-apoptotic members, such as *BCL2*, prevent the onset of apoptosis by suppression of pro-apoptotic proteins, increasing cell survival, and promoting the cell cycle process (6, 7). 

The specific feature of nanoparticles that increased their application in medical sciences as drug delivery systems is the high ratio of surface to volume. Carbon nanostructures have unique physicochemical properties that make them an important family in the field of nanomedicine (8). Graphene is a two-dimensional carbon allotrope consisting of hexagonal structures like a honeycomb with the thickness of an atom. This transparent nanoparticle has become one of the hottest topics in the fields of physics, chemistry, and nanotechnology. Graphene is the hardest and thinnest substance ever produced by human beings (9). Graphene oxide (GO) is obtained from complete oxidation and peeling of graphite, which includes a wide range of reactive oxygen groups containing epoxy, hydroxyl, carbonyl, and carboxyl groups. These reactive groups at the surface of GO make it soluble and increase the affinity to bond with other substances and drugs (10, 11).

Quinoline is an aromatic nitrogen compound that has various derivatives with a wide application range (12). One of these derivatives is 8-hydroxyquinoline (8-HQ), which has the ability to form complexes with bivalent metallic ions and has the potential to interfere with cancerous cellular pathways providing anti-cancer properties (13). 8-HQ and its derivatives have been used in medicine as anticancer, antioxidant, antimicrobial, anti-inflammatory, and anti-neurodegenerative agents. Its anticancer properties include prevention of cancer cell proliferation and tumor angiogenesis and induction of apoptosis in cancerous cells (13, 14).

The anticancer properties of 8-HQ and the increasing use of GO nanocomposites as drug carriers as well as its anti-cancerous properties (15), led us to use GO as the carrier of 8-HQ and investigate the anti-cancer properties of 8-HQ-coated GO by evaluating the toxicity and apoptosis-induction capability of this nanocomposite on breast cancer cells compared with normal breast cells.

## Materials and Methods

***Synthesis of nanocomposites***

GO was prepared by oxidizing graphite powder according to the improved Hummers method (16). In summary, 0.5 g of graphite powder (Sigma Aldrich, USA) and 0.3 g of sodium nitrate (Sigma Aldrich, USA) were added to 16.5 ml cold sulfuric acid (Merck, Germany). The suspension was blended for a period of 20 min at a rate of about 150 *RCF*. After reaching the temperature to 0 °C in an ice bath, 1.5 g of potassium permanganate (Sigma Aldrich, USA) was added gradually over a period of 30 min to prevent sudden temperature rise. 30 ml of deionized water was slowly added to the suspension and 2 ml of hydrogen peroxide (Merck, Germany) was added dropwise. The suspension was washed several times with hydrochloric acid (Merck, Germany) and ethanol and centrifuged at 1800 *RCF*. The product was let to dry.

8-HQ used in this study was activated and bonded to mesoporous silica SBA-15, according to the protocol presented by Badiei *et al.* (17). To bind GO to silica-bound 8-HQ, 20 mg of GO was dissolved in 5 ml of dimethylformamide (Merck, Germany) and 50 mg of 4-dimethylaminopyridine (Merck, Germany) and 50 mg of N,N’-dicyclohexylcarbodiimide (Merck, Germany) were added and mixed at room temperature for 30 min. 30 mg of SBA-15-bounded 8-HQ was dissolved in 3 ml of dimethylformamide (Merck, Germany) in the presence of 1 ml of 10% hydrochloric acid (Merck, Germany). Then, the GO mixture was added and mixed overnight at room temperature. The solid phase was separated by centrifugation and washed twice with dimethylformamide and once with methanol (Merck, Germany). The resulting deposition was dissolved in 10 ml of deionized water and sonicated for 3 min using an ultrasonic system (Elma Ultrasonic, Germany). The detailed synthesis procedure, as well as the physicochemical properties of the nanocomposite such as its TEM and SEM view, were published previously (18).

***Treatment of breast cell lines with nanocomposite***

1×10^4^ cells of each MCF-7 breast cancer cell line (ATCC, USA) and normal breast epithelial cell line (MCF-10A) (ATCC, USA) were cultured triplicate in 200 μl of DMEM (Gibco, USA) containing 10% FBS (Gibco, USA) and 1% penicillin-streptomycin (Gibco, USA) in two 96-wells plates (SPL, Korea) and nanocomposites were added at concentrations of 0.031, 0.062, 0.125, 0.25, and 0.5 mg/ml. There was no addition of the nanocomposite in the control wells. Plates were kept at a cell-culture incubator for 12, 24, or 48 hr.

***MTT and viability assay***

Twenty microliters of MTT solution (Invitrogen, USA) with a concentration of 0.5 mg/ml was added to each well and plates were incubated for 4 hr in the dark. The supernatant was then discarded and 150 μl DMSO (Merck, Germany) was added to the solution. The optical density of the produced dye at a wavelength of 570 nm was measured using an ELISA reader (BioTek, USA) and the percentage of live cells was calculated. The viability test was performed using trypan blue dye (Sigma, UK) for the cells treated with 0.5 mg/ml of the nanocomposite for 48 hr. The percentages of dead and live cells were measured by counting cells with Neobar Lam (HBG, Germany) and a light microscope (Zeiss, Germany).

***Study of apoptosis in nanocomposite-treated cell lines***

*Evaluation of BAX, BCL2, P53 and P21 gene expressions*

The cell lines were treated with a concentration of 0.5 mg/ml of the nanocomposite for 48 hr. The cells were transferred to RNase-free microtubes and centrifuged for 5 min at 250 *RCF* and the supernatant was discarded. Total RNA was extracted using an RNA extraction kit (RNeasy mini kit Qiagen, Germany) according to the manufacturer’s instructions. The quantity and quality of the extracted RNA were determined by measuring the absorbance of the RNA samples at 260, 230, and 280 nm using a PicoDrop (Spectrometer pico-drop, UK), and also 2 μl of RNA was electrophoresed on agarose gel 1%. Synthesis of cDNA using a cDNA synthesis kit (RevertAid first-strand cDNA synthesis, Fermentas, Thermo Fisher Scientific, USA) was performed according to the kit instructions. Briefly, 9 μl of extracted RNA plus 1 μl of random hexamer primer, 4 μl of reaction buffer, 1 μl of RNase inhibitor, 2 μl of dNTP (10 mM), and 1 μl of reverse transcriptase enzyme were added together and supplemented with nuclease-free water to reach 20 μl. Microtubes were placed in a thermal cycler (PeQlab, Germany) for 5 min at 25 ^°^C, 60 min at 42 ^°^C, and 5 min at 70 ^°^C. The cDNA was dissolved in nuclease-free water and kept at -20 ^°^C.

For qRT-PCR reaction, the *β-actin *gene was used as a reference gene and the primers used in this study are shown in Table 1 (19). 28 ng of the synthesized cDNA was added to a mixture containing 4 μl Quantifast SYBR Green master mix (Qiagen, Europe) and 0.5 μM of each of forward and reverse primers and the final volume was supplemented to reach 20 μl. The samples were then placed in a rotor gene 6000 real-time rotary analyzer (Corbett life science, Australia) and after 10 min of initial denaturation at 95 ^°^C, samples were amplified during 40 cycles each comprising 30 sec at 95 ^°^C, 30 sec at 59 ^°^C, and 45 sec at 72 ^°^C. The Livak method was used to analyze gene expression (20).

*Apoptosis evaluation by Annexin-V/PI assay*

The cell lines were treated with 0.5 mg/ml nanocomposite for 48 hr. The cells were washed twice with cold PBS and dissolved in 5 ml of binding buffer containing 5 μl of each of annexin-V-FITC (BD, USA) and PI (BD, USA) with the concentration of 250 μg/ml. After 15 min of incubation in the dark, samples were read using a flowcytometer (FACSCalibur, BD, USA).

***Statistical analyses***

All tests were performed in triplicate. The Flowjo (Treestar) software was used to analyze and show the flow cytometric results. SPSS (version 19) and PRISM 7 software packages were used for statistical analysis and *P*-value<0.05 was considered significant. The data were described as mean with standard deviation. Analysis of variance (One-way ANOVA) was used to compare more than two quantitative variables.

## Results

***Evaluation of nanocomposite cytotoxicity by MTT and viability assays***

**Figure 1:**
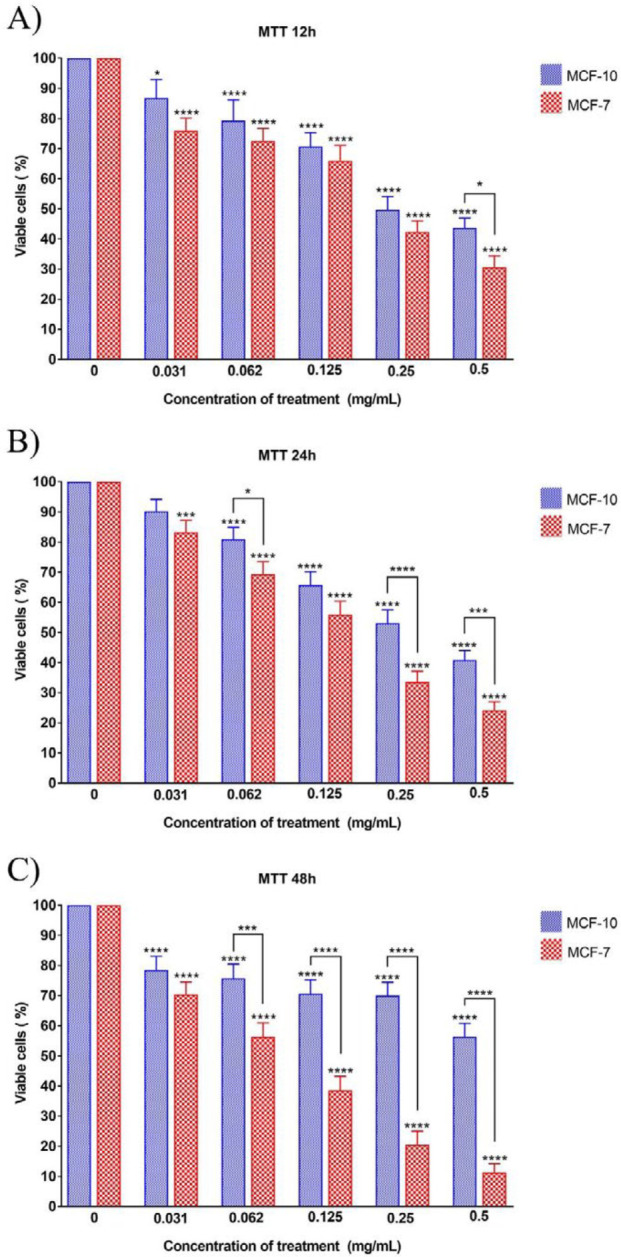
Bar chart of viable cell lines after exposure to five concentrations of nanocomposite for 12, 24, and 48 hr. The stars on each column indicate a significant difference compared with the related control (untreated cells) and the stars on the lines indicate a significant difference between the different treated cell lines. *P*-value<0.05:*, *P*-value<0.01:**, *P*-value<0.001:***, *P*-value<0.0001:****

MCF-7 and MCF-10 cell lines were treated with different concentrations of the nanocomposite in three periods of 12, 24, and 48 hr, and MTT assay was performed to study the toxicity of the nanocomposite. The percentages of live cells of each cell line at each concentration of drug treatment for 12, 24, and 48 hr are presented in Figure 1.

**Figure 2:**
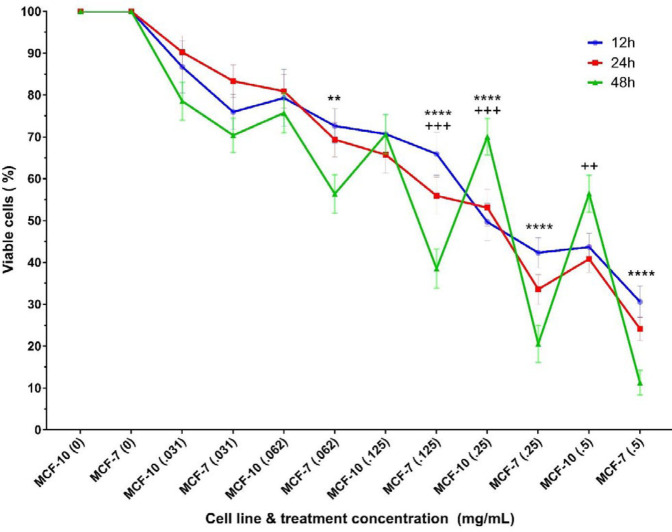
Trend of nanocomposite cytotoxicity on cell lines after exposure to five concentrations of nanocomposite for 12, 24, and 48 hr. The blue, red, and green lines are representative for 12, 24, and 48 hr treatments, respectively. Stars indicate significant differences in the viability of cells between 12 hr and 48 hr exposure to the nanocomposite. The plus signs indicate a significant difference in the viability of cells between 24 hr and 48 hr exposure to the nanocomposite. *P*-value<0.01:**, *P*-value<0.001:***, *P*-value<0.0001:****

The results show that the percentages of viable cells in both normal and cancer cell lines treated with any concentration (0.031, 0.062, 0.125, 0.25, and 0.5 mg/ml) of the nanocomposite for any period (12, 24, and 48 hr) has significantly decreased compared with the related untreated control. The decreasing rate of cell viability was dose-dependent such that with increasing nanocomposite concentration, the mortality rate of cells also increased. Nevertheless, the results show higher cell death rate in the MCF-7 cell line, compared with the MCF-10 cell line, which is statistically significant at the concentration of 0.5 mg/ml of the nanocomposite. In 24 hr treatment, the mortality rates of the MCF-7 cell line at concentrations of 0.062, 0.25, and 0.5 mg/ml of the nanocomposite were significantly lower than those of MCF-10 cell line. Most differences in cell death are seen after 48 hr of treatment with different concentrations of nanocomposites such that there are significant lower mortality rates in MCF-7 cancer cell lines compared with normal MCF-10 cell lines at all concentrations of treatment with the nanocomposite, except for the concentration of 0.031 mg/ml. Figure 2 shows the trend of cell viability based on treatment time and concentration. As illustrated, increasing the time of treatment in almost all cases decreased cell viability. Moreover, in some treatment concentrations, 48 hr treatment significantly decreased cell viability, compared with 12 hr and 24 hr treatments. No significant difference was found between the viability percentages of 12 hr and 24 hr treated cells.

**Figure 3:**
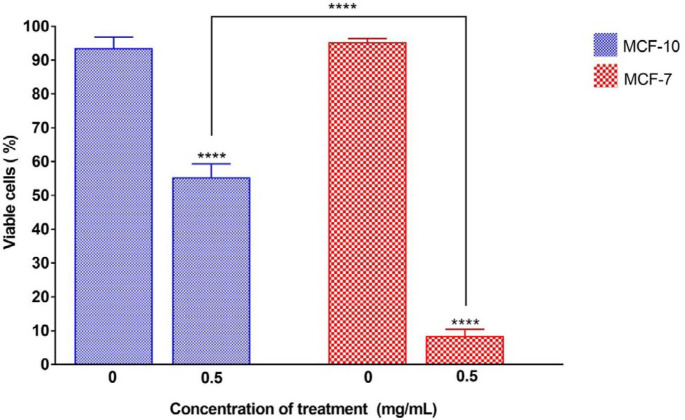
Percentage of viable cell lines after 48 hr of exposure to 5 mg/ml of the nanocomposite. The stars on each column show a significant difference compared with the related untreated control, and the interconnected lines indicate a significant difference between the different treated cell lines. *P*-value<0.0001:****

To show the maximum cytotoxic effect of the nanocomposite, all the remaining experiments were performed using 48 hr exposure of cells to 0.5 mg/ml nanocomposite. The results of calculating the percentage of live cells after 48 hr exposure to 0.5 mg/ml of nanocomposite using trypan blue viability assay are shown in Figure 3. The results indicate that the percentages of viable cells in both cell lines decreased significantly compared with their untreated control samples. Also, there were significant differences in the percentages of cell death among breast normal and cancer cell lines. The MCF-7 cell line had significantly higher cell death (92%) compared with cell death in the normal MCF-10 cell line, which was about 45%.

***Evaluation of apoptosis in nanocomposite-treated cell lines***

*Measurement of Bax, BCL2, P53, and P21 gene expressions*

**Figure 4.**
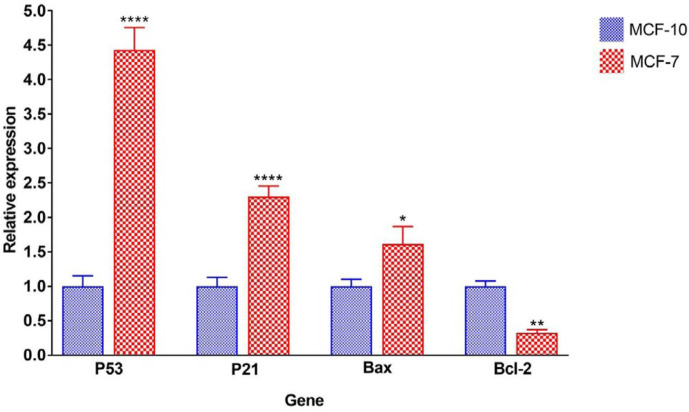
Relative expression of P53, P21, Bax, and Bcl-2 genes in MCF-10 and MCF-7 cell lines treated with 5 mg/ml of nanocomposite. The stars on each column showed a significant difference compared with the control (MCF-10 cell line) and the stars on the lines indicate a significant difference between the various treated cell types. *P*-value<0.05:*, *P*-value<0.01:**, *P*-value<0.001:***, *P*-value<0.0001:****, n.s.: not significant

The optical absorption ratios of 260/280 and 260/230 nm were determined and samples with ratios between 1.8-2 were selected. Observation of the 18S and 28S bands, and the double strength of 28S to the 18S band, as well as the absence of smear on agarose gel, showed the purity and integrity of RNA. After confirming the quantity and quality of extracted RNA, cDNA was synthesized from RNA and expression of *P53*,* P21*,* BAX, *and* BCL2* genes was measured using the realtime-PCR method. The *β-actin* gene was used as the reference gene. Experiments were carried out in triplicate and the results were analyzed using the Livak formula. The expression level of the genes in MCF-10 cells was assumed as the reference and the expression of the genes in the MCF-7 cancer cell line was compared with those of MCF-10 and shown in Figure 4.

The results indicate that the expression of *P53*, *P21,* and *BAX* genes was significantly increased in MCF-7 cancer cell line compared with MCF-10. The expression level of *P53*, *P21,* and *BAX* genes in the MCF-7 cell line was approximately 4.4, 2.3, and 1.6 folds higher than those in the MCF-10 cell line, respectively. In contrast, the expression level of the *BCL2* gene in the MCF-7 cell line was significantly reduced to one-third of the *BCL2* expression level in the normal MCF-10 cell line.

*Apoptosis evaluation by Annexin-V/PI assay*

**Figure 5:**
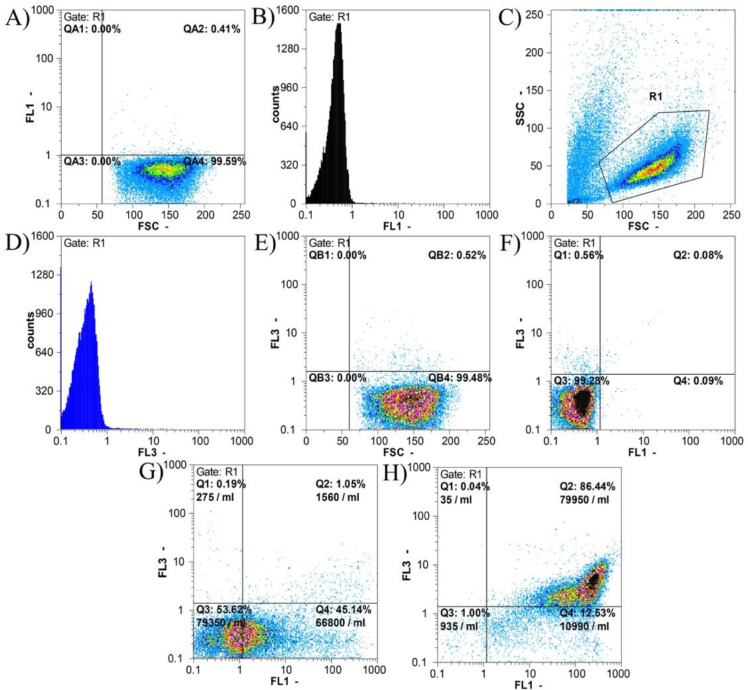
Flow cytometric results of apoptosis in MCF-10 and MCF-7 cell lines treated with 0.5 mg/ml of nanocomposite for 48 hr using the Annexin-V-FITC/PI assay. A-F) Flowcytometric results on the normal MCF-10 cell line without nanocomposite treatment. In Figures A and B, the percentage and number of events counted in the FL1 channel, which are related to the Annexin-V-FITC color, are displayed. The method of selecting the population (gating strategy) is shown in Figure C. Figures E and D show the number of events counted in the FL3 channel, which are related to the PI color. In Figure F, based on both Annexin-V-FITC (FL1) and PI (FL3) colors, a quadrant chart is drawn that shows each of the cell groups. In Figures F to I: Q1 is the percentage of necrotic cells, Q2 is the percentage of cells in the late phase of apoptosis, Q3 is the percentage of cells in the early phase of apoptosis and Q4 represents the percentage of live cells. According to the gating strategy shown in section C, the results of apoptosis in other cell lines were calculated. Figures G, and H respectively show the results of apoptosis examination in MCF-10 and MCF-7 cell lines treated with 0.5 mg/ml nanocomposite for 48 hr

To determine whether the reduction of viable cell lines following treatment with the nanocomposite is apoptosis-mediated, the release of phosphatidylserine at the cell surface after 48 hr of treatment with 0.5 mg/ml of the nanocomposite was analyzed by Annexin-V-FITC/PI staining and flow cytometric analysis. The results are shown in Figure 5.

As shown in Figure 5, 99.28% of untreated MCF-10 cells were alive and only 0.56% of cells were necrotic. 0.09% and 0.08% of cells were in the early and late stages of apoptosis, respectively; cumulatively 0.17% of the cells were apoptotic.

After treatment of MCF-10 cells with 0.5 mg/ml of nanocomposite for 48 hr, 53.62% of the cells were alive and only 0.19% of the cells were necrotic. The apoptotic cell population comprised 46.19% of the cells from which 45.14% were in the early and 1.05% of the cells were in the late apoptosis stage.

The maximum apoptosis induction after treatment with 0.5 mg/ml of nanocomposite for 48 hr was seen in the MCF-7 cell line in which only 1% of the cells were alive and 0.04% of the cells were necrotic. The rest of the cells (98.97%) underwent apoptosis divided into early (12.53%) and late (86.44%) phases.

**Figure 6:**
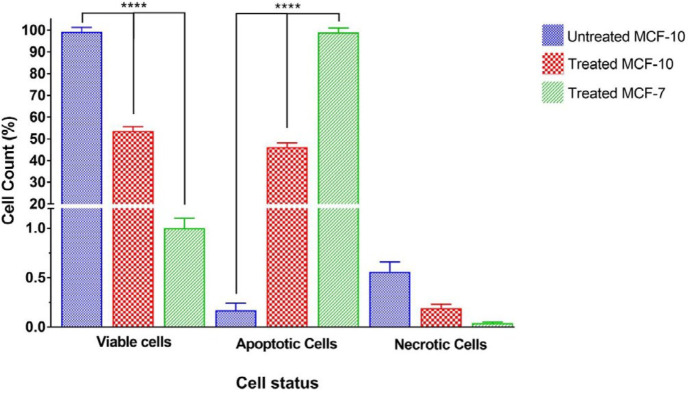
Comparison of percentages of live, apoptotic, and necrotic cell lines after treatment with 0.5 mg/ml concentration of nanocomposite for 48 hr. The stars on the lines show the significant difference between the various treated cell lines. P-value<0.0001:****

Comparison of apoptotic, necrotic, and viable cell lines that were exposed for 48 hr to 0.5 mg/ml nanocomposite is shown in Figure 6. As shown, the percentage of live cells is significantly different across both treated cell lines and also with untreated MCF-10. The untreated MCF-10 cell line has the highest number of living cells and among the treated cell lines, MCF-7 had significantly lower living cells. Also, the percentage of apoptotic cells was significantly different between both treated cell lines, as well as the untreated MCF-10 cell line. The untreated MCF-10 cell line had the lowest number of apoptotic cells and also among the treated cell lines, MCF-7 had significantly higher apoptosis. There was no significant difference in the results of necrotic cells between the three groups.

**Table 1:** Primers used in the study of the apoptosis-related genes expression

Reference	Product size (bp)	Ta	Nucleotide sequence 5’ → 3’	Primer
**(** 18 **)**	293	60C	5′-ACCAAG CTGAGCGA GTGTC-3′	BAX (F)
**(** 18 **)**	293	60C	5′-ACAAAGATGGTCACGGTCTGCC-3′	BAX (R)
**(** 18 **)**	415	60C	5′-ACCAAG CTGAGCGA GTGTC-3′	Bcl-2 (F)
**(** 18 **)**	415	60C	5′-ACAAAGATGGTCACGGTCTGCC-3′	Bcl-2 (R)
**(** 18 **)**	473	64C	5′-GTTTCCGTCTGGGCTTCTTG-3′	P53 (F)
**(** 18 **)**	473	64C	5′-CCTGGGCATCCTTGAGTTCC-3′	P53 (R)
**(** 18 **)**	517	60C	5′-CTCAGAGGAGGCGCCATG-3′	P21 (F)
**(** 18 **)**	517	60C	5′-GGGCGGATTAGGGCTTCC-3′	P21 (R)
**(** 18 **)**	350	60C	5′-AACCGCGAGAAGATGACCCAGATCATGTTT-3′	β-actin (F)
**(** 18 **)**	350	60C	5′-AGCAGCCGTGGCCATCTCTTGCTCGAAGTC-3′	β-actin (R)

F: Forward; R: Reverse; bp: Base pair

## Discussion

This study showed that the 8-HQ-coated GO had high toxicity for MCF-7 breast cancer cell line, while it was very low toxic for MCF-10 normal breast cells. It might indicate the relative specific toxicity of nanocomposite for cancer cell lines. It was also observed that the effects of the nanocomposite toxicity are dose- and time-dependent.

It has been reported that binding GO to other nanoparticles or chemicals increases its efficacy. For instance, binding SN38 and doxorubicin (DOX) to GO increases solubility, biocompatibility, distribution in tumor tissue and anti-cancer effects (21, 22). One of the biggest problems of GO application is aggregation in physiological solutions which could be solved by binding to other nanomaterials (23). In this study, 8-HQ was coated with a mesoporous silica SBA-15 and then coated with GO.

The toxicity of carbon nanoparticles such as graphene, GO, and carbon nanotubes to different classes of cancerous and normal cells, has been shown and pointed to the role of shape and structure of nanoparticles in their toxicity (15, 24, 25).

Studies have also shown the synergistic effects of GO with other anticancer drugs (26, 27), which was the reason for binding 8-HQ to GO in our study. One of the considerable drawbacks of our study is the lack of individual investigation of silica, GO, and 8-HQ anti-cancer effects separately and comparing with the 8-HQ-coated GO. In this case, we could clearly show the effects of each alone and the synergistic effects of the combination. However, according to the past studies, GO and SBA-15 mesoporous silica have low cytotoxicity effects on MCF-7 or other cell lines introducing them as biocompatible drug delivery systems for anti-cancer treatment (28-30). On the other hand, 8-HQ has been shown to have cytotoxic effects on MCF-7 cell line by itself and in combination with other anti-cancer agents (31).

So far, various nanomaterials have been used as carriers of anticancer drugs (32, 33), of which GO showed more advantages in loading drugs including doxorubicin (34), paclitaxel (27), methotrexate (35), and other drugs in various cancers. Also, the specificity and increased cytotoxicity of GO-bounded doxorubicin in comparison with doxorubicin are shown by MTT and similar assays on cancer cells, including MCF-7 (26, 36). In many of these studies, the effects of GO on normal cells have been reported very rarely, indicating the relative specificity and greater effect of this substance on cancer cells, although the exact mechanism of this difference in effect is still unknown (15). Fiorillo *et al.* (37) showed that GO specifically inhibits the proliferation of cancer cell stem cells (CSCs) in various cancers and is not toxic to the normal pluripotent stem cells, but induces their differentiation into different cell types (37, 38). Given that the cells used in this study were cancer cell stem cells (CSC), one of the causes of the high toxicity of nanocomposites could be the induction of cell differentiation and cell death in these cell lines. Some derivatives of quinolines including 8HQ have anti-cancer properties due to their ability to chelate the bivalent ions needed to grow cancer (39, 40). In our study, the sensitivity of the breast cancer cell line to the 8HQ nanocomposite has been well illustrated. Studies on the MCF-7 cell line provided evidence on the specific effect of quinoline derivatives and showed that breast cancer cells are susceptible to these compounds in the MTT assay (41, 42). 

In the present study, the expression of pro-apoptotic genes in the MCF-7 treated cell line with nanocomposite was higher, compared with control (MCF-10), indicating the effects of nanocomposite on the induction of apoptosis in this cancer cell line. *P21* is one of the crucial genes in inducing cell cycle arrest (6) and increasing its expression in the cancer cell line can increase the probability of nanocomposite-induced cell cycle arrest which requires more confirmatory tests. On the other hand, expression of the anti-apoptotic *BCL2* gene in the treated breast cancer cell line has significantly decreased compared with control cells representing a probable reduction in survival signals in these cancer cells. The Annexin-V/PI assay also indicated that severe apoptosis was induced in breast cancer cells following treatment with nanocomposites, which was significantly higher than that of MCF-10. It might show that the MCF-7 cells are more susceptible to apoptosis induction via 8HQ-GO nanocomposites compared with MCF-10.

The previous studies have shown that cell cycle arrest and apoptosis are mechanisms of cytotoxicity of quinoline derivatives and GO (15, 43). Quinoline derivatives inhibit the signaling pathway of PI3K-Akt-mTOR leading to cell cycle arrest and apoptosis induction in MCF-7 (42). In this study, we observed that the 8HQ-containing nanocomposite has much lower toxicity on normal breast epithelial cells than on cancer cells. In similar studies, 8HQ-titanium complexes in the MTT assay exhibited high cytotoxicity characteristics against BEL-7404, HepG2, NCI-H460, T-24, and A549 cancer cells while little toxicity was found on HL-7702 normal liver cell line. It has also been pointed out that these complexes inhibit cell cycle progression and induce mitochondrial apoptosis (44).

## Conclusion

Conclusively, the 8HQ-coated GO has relatively specific toxicity for MCF-7 breast cancer cell line, while it has much lower toxicity to normal breast epithelial cells. One of the main mechanisms of this cytotoxicity is the induction of apoptosis such that the nanocomposite induced molecular and cellular apoptosis in cancer cells whereas low apoptosis was observed in treated normal breast epithelial cells. The point that should not be overlooked is that all these results were obtained by *in vitro* study on cell lines. The *in vivo* antitumor effects of this nanocomposite should also be investigated. Interestingly, the *in vivo* anti-tumor properties of 8HQ-2-quinoline carbaldehyde were studied on nude male rats bearing hepatocellular carcinoma xenograft tumor (Hep3B) and showed that intraperitoneal injections of nanocomposite have completely stopped the growth of the tumor and caused no tissue damage on the vital organs of the mouse (45). It is suggested to investigate molecular and cellular mechanisms more precisely, including cell proliferation, cell cycle, migration, invasion, metastasis, and angiogenesis following nanocomposite treatment on these cell lines and other cancerous or normal cells to evaluate the specificity of the nanocomposite functions.
